# The *miR-15a-5p-XIST-CUL3* regulatory axis is important for sepsis-induced acute kidney injury

**DOI:** 10.1080/0886022X.2019.1669460

**Published:** 2019-10-28

**Authors:** Guanhua Xu, Lujiao Mo, Channi Wu, Xiaoyuan Shen, Hongliang Dong, Lingfeng Yu, Ping Pan, Kanda Pan

**Affiliations:** a Department of Intensive Care Unit (ICU), The First People's Hospital of Xiaoshan District, Hangzhou, Hangzhou, China;; b Department of Gastroenterology, Zhejiang Xiaoshan Hospital, Hangzhou, China;; c Department of General Medicine, The First People's Hospital of Xiaoshan District, Hangzhou, Hangzhou, China

**Keywords:** Acute kidney injury, differentially expressed miRNAs, enrichment analysis, competing endogenous RNA, regulatory network analysis

## Abstract

**Background:** Acute kidney injury (AKI) refers to a sudden loss of renal function. This study was performed to identify the key RNAs acting in the mechanism of sepsis-induced AKI.

**Methods:** Microarray dataset GSE94717 (including six sepsis-induced AKI samples and three control samples) was downloaded from Gene Expression Omnibus database. Differentially expressed miRNAs (DE-miRNAs) were identified. The miRNA targets were predicted and enrichment analysis was performed. Protein-protein interaction (PPI) and competing endogenous RNA (ceRNA) regulatory networks were constructed. Mouse podocytes were treated with lipopolysaccharide (LPS), following by cell viability and PCR analysis. Cellular apoptosis and the ceRNA network were validated.

**Results:** Thirty-one common DE-miRNAs (two up-regulated and 29 down-regulated) by AKI versus control and male AKI versus control were identified. We found the targets of *miR-15a-5p*, *miR-15b-5p*, and *miR-16-5p* were involved in mTOR signaling pathway, and those of *miR-29b-3p and miR-16-5p* were enriched in PI3K-Akt signaling pathway. RNAs including *miR-15b-5p*, *miR-15a-5p*, *miR-107*, *XIST*, *miR-16-5p*, and cullin 3 gene (*CUL3*) were included in the ceRNA regulatory network. The downregulation of *miR-15a-5p* and *miR-15b-5p* and the upregulation of lncRNA *XIST* and *CUL3* gene were validated using qPCR. The *miR-15a-5p-XIST-CUL3* regulatory axis was identified and was validated. We confirmed that LPS inhibited the growth of mouse podocytes and seven of the ten miRNAs, but upregulated *XIST* and *CUL3*. Transfection analysis showed XIST siRNA enhanced LPS-induced MPC5 cell apoptosis and *miR-15a-5p* inhibitor reserved it, so did as *CUL3* overexpression for *miR-15a-5p* mimics.

**Conclusion:** The *miR-15a-5p-XIST-CUL3* regulatory axis was related to the pathogenesis of sepsis-induced AKI.

**Highlights**
- Totally, 31 miRNAs were dysregulated between disease and control groups.
- *MiR-15a-5p*, *miR-15b-5p*, and *miR-16-5p* were involved in mTOR signaling pathway.
- *MiR-16-5p* and *miR-29b-3p* were implicated in PI3K-Akt signaling pathway.
- The miR-15a-5p-XIST-CUL3 axis was critical for sepsis-induced AKI.

## Introduction

Acute kidney injury (AKI) is a sudden loss of renal function that happens in less than seven days [1]. It can be induced by various processes, and the most common causes are dehydration, sepsis, and nephrotoxic drugs [2,3]. AKI is usually diagnosed based on increased blood and urea nitrogen, or insufficient urine produced by the kidneys [4]. The complications of AKI include high potassium content, metabolic acidosis, variation of fluid balance, and uremia [5]. Sepsis-induced AKI occurs in 40% patients with sepsis, namely septic AKI [6]. Recent studies demonstrated that patients with AKI may have increased renal blood flow, tubular injury but not necrosis and apoptosis [6,7]. Therefore, the mechanisms of AKI are complex and should be deeply explored to improve the therapies of the disease.

MicroRNA (miRNA) can cause gene silencing by binding mRNA, while competing endogenous RNA (ceRNA, such as long noncoding RNA (lncRNA), mRNA, circular RNA (circRNA), pseudogene transcript, et al) can mediate gene expression by competitively binding miRNA using microRNA response elements (MREs) [8,9]. For instance, Sirtuin 3 (*SIRT3*) protects against mitochondrial damage in sepsis-induced AKI through suppressing the NLR family, pyrin domain-containing 3 (*NLRP3*) inflammasome, reducing reactive oxygen species (ROS) production, decreasing interleukin-1β (*IL1B*) and *IL18* expression, and weakening oxidative stress [10,11]. LncRNA HOX transcript antisense RNA (*HOTAIR*) has a high expression in sepsis-induced AKI, which facilitates the apoptosis of HK-2 cells in AKI via the *miR-22*/high mobility group box 1 (*HMGB1*) pathway [12]. Through repressing c-Jun N-terminal kinase (JNK)/nuclear factor-κB (NF-κB) pathway and binding to tumor necrosis factor-α (*TNF-α*), lncRNA plasmacytoma variant translocation 1 (*PVT1*) may enhance inflammatory response in lipopolysaccharide (LPS)-induced septic AKI [13]. In addition, overexpressed lncRNA nuclear paraspeckle assembly transcript 1 (*NEAT1*) may worsen AKI via stimulating NF-κB pathway and regulating *miR-204*, therefore, *NEAT1* may have impressive roles in sepsis-induced AKI [14]. Although the above studies have explored the RNAs involved in sepsis-induced AKI, the pathogenesis of this disease have not been entirely reported.

In the current study, the miRNA expression profile of sepsis-induced AKI was downloaded and analyzed. Through differential expression analysis, miRNA-target prediction, enrichment analysis, and network analysis, the crucial RNAs and ceRNA regulatory relationships were identified in sepsis-induced AKI. This study might further reveal the pathogenesis of the disease and provide theoretical support for its clinical treatment.

## Materials and methods

### Microarray data

The miRNA microarray dataset, under GSE94717 [https://www.ncbi.nlm.nih.gov/geo/query/acc.cgi?acc=GSE94717, platform: GPL19449Exiqon miRCURY LNA microRNA Array, 7th generation REV-hsa, mmu & rno (miRBase v18.0)] was downloaded from Gene Expression Omnibus (GEO) database. GSE94717 consisted of 15 blood samples collected from 6 patients with G- sepsis-induced AKI, 6 patients with G- sepsis-non AKI and 3 healthy controls. The data from samples from patients with G- sepsis-induced AKI (*n* = 6, mean age = 64.17 years old, two females and four males) and from healthy controls (*n* = 3, mean age = 60 years old, two females and one male) were selected for the further analysis.

### Data preprocessing and differential expression analysis

The matrix data of the microarray dataset was acquired and subjected to background correction and normalization using the R package limma (version3.10.3, http://www.bioconductor.org/packages/2.9/bioc/html/limma.html) [15]. Subsequently, the expression matrix was divided into sepsis-induced AKI group and control group, and the significance of *p* values of expression difference was calculated based on the unpaired *t*-test in limma package [15]. The differentially expressed miRNAs (DE-miRNAs) were identified with the thresholds of |log fold change (FC)|≥1 and *p* values <.01.

### miRNA-target prediction and enrichment analysis

The target genes of the DE-miRNAs were predicted using teh miRWalk2.0 tool [16] (http://zmf.umm.uni-heidelberg.de/apps/zmf/mirwalk2/). To ensure the accuracy of target prediction, the miRNA-target pairs included in at least 7 of miRWalk, miRanda, miRDB, miRMap, miRNAMap, RNA22, Targetscan, and mirbridge databases were screened. The miRNA-target regulatory network was constructed using Cytoscape software (version 3.2.0, http://www.cytoscape.org) [17].

Bioinformatics enrichment was performed for the genes included in the miRNA-gene pairs. Gene Ontology (GO), including cellular component (CC), biological process (BP), and molecular function (MF) categories [18] and Kyoto Encyclopedia of Genes and Genomes (KEGG) pathway [19] enrichment was conducted combined with DAVID online tool [20] (version 6.8; https://david-d.ncifcrf.gov/). Meanwhile, the R package clusterprofiler [21] (https://bioconductor.org/packages/release/bioc/html/clusterProfiler.html) was utilized to perform KEGG enrichment analysis for the miRNAs in the miRNA-target regulatory network with the number of target genes ranked in the top 10. The significant thresholds for selecting the results of enrichment analysis were set as gene count ≥ 2 and *p* values < .05.

### Protein-protein interaction (PPI) network analysis for the target genes

The interactions among the genetic productions of the targets were identified in STRING database [22] (version 10.0; http://string-db.org/; combined score > 0.4). PPI network was visualized using the Cytoscape software [17]. To obtain the key target genes, the network topology property index Degree Centrality was used to analyze the scores of network nodes. The higher the node score, the more important the location of the node was in the network. The significant network modules were screened using the MCODE plug-in [23] in Cytoscape software, with the threshold of score > 5.

### CeRNA regulatory network analysis

The miRNA-lncRNA pairs involving the DE-miRNAs were screened in starBase database [24] (version 2.0, http://starbase.sysu.edu.cn/), with the thresholds of low stringency ≥ 1 and number of cancer types ≥ 1. The lncRNA and mRNA regulated by the same miRNAs were screened from the miRNA-mRNA pairs and miRNA-lncRNA pairs, namely the miRNA-lncRNA-mRNA or ceRNA pairs. ceRNA regulatory network was visualized using Cytoscape software [17].

### Patient collection

A total of five patients (male = 4 and female = 1, aged 45.6 ± 6.9 years old) were collected from Department of ICU, the first people's hospital of Xiaoshan District, Hangzhou, during February 2019 to July 2019. Five sex- and age-matched healthy controls (47.1 ± 8.2 years old), without known diseases, were collected from our hospital. The fasting peripheral blood samples were collected from all patients and healthy controls. Blood samples were prepared and RNA was isolated and stored at −20 °C before analysis. The human experiments were approved by the Ethics Committee of the First People's Hospital of Xiaoshan District, Hangzhou. Written informed consents were obtained from all participants before blood sampling.

### Cells and LPS induction

Mouse podocytes (MPC5) were purchased from the Cell Bank of the Chinese academy of sciences (Shanghai, China). Cells were maintained in RPMI-1640 (Invitrogen, Shanghai, China) supplemented with 10% FBS (Invitrogen) at 37 °C, 5% CO_2_. MPC5 were treated with 100 ng/mL LPS (Sigma-Aldrich) for 48 h [25].

### Cell proliferation assay

Cell viability was tested using the Cell Counting Kit 8 (CCK8) assay kit (Beyotime, Shanghai, China). Cells were harvested at 0, 12, 24, and 48 h post LPS induction and then incubated in CCK8 solution for 2 h. Cell viability was detected using a microplate reader (Bio-Rad Labs, Sunnyvale, CA) and the optical density at 450 nm was detected. Each experiment was performed in triplicate.

### Dual-luciferase reporter assay

The interactions between lncRNA and miRNA and between miRNA and target were predicted using the LncBase Predicted v(0).2 (http://carolina.imis.athena-innovation.gr/diana_tools/web/index.php) and miRTarBase (http://mirtarbase.mbc.nctu.edu.tw/php/detail.php), respectively. The interaction was validated using dual-luciferase reporter system. The luciferase vectors containing wild type (WT) and mutant (MUT) binding sites of miRNAs in the 3′ UTR regions of XIST and DUL3 genes were constructed using psiCHECK-2 expression vector (Promega, USA) [26]. Cell transfection into MPC5 cells was performed using Lipofectamine 2000 regents (Invitrogen), following the manufacturer’s instructions.

### Cell transfection

MPC5 (1 × 10^5^ cells/well) were seeded into 24-well plates, and then transfected with siRNAs targeting *XIST*, *miR15b-5p* mimics, inhibitors, and the scramble sequences (NC; GenePharma, Shanghai, China) for 6 h. For the overexpression of *CUL3* gene, cells were transfected with *CUL3*-overexpressing (OE-CUL3) plasmids constructed by cloning the full length of human *CUL3* gene coding region into pcDNA3.1 vectors (Genechem). Empty pcDNA3.1 was used as control for OE-CUL3 transfection. Cell transfections were conducted using the Lipofectamine 2000 (Invitrogen) according to the manufactures’ instruction. Then MPC5 cells were treated with 100 ng/mL LPS (Sigma-Aldrich) for 48 h. Cell proliferation was detected after LPS treatment.

### Cellular apoptosis

LPS-induced MPC5 cell apoptosis was detected using Annexin V/PI double staining (BD Biosciences, San Jose, CA, USA). Transfected cells (5 × 10^5^ cells/ml) were placed into 6-well plates and then treated with LPS as previously reported. Cells were harvested and then digested into single-cell suspensions, which were then incubated with Annexin V-FITC/PI staining solutions (BD Biosciences) and then detected using a BD FACS Calibur™ flow cytometry (BD Biosciences). Each experiment was performed in triplicate.

### Real-time PCR analysis

Cellular and blood RNAs were extracted using TRIzol reagent (Invitrogen). Reverse transcription of miRNA and mRNA was performed with reverse transcription and a DBI Bestar qPCR RT Kit (DBI Bioscience, Shanghai, China), respectively, following the manufacturer’s instructions. Primers were synthetized by Sangon (Shanghai, China; Table 1). Applied Biosystems 7500 Fast Real-Time PCR System (Applied Biosystems, Foster City, CA, USA) was employed for the PCR amplification. *GAPDH* was used as reference gene for mRNA and lncRNA, and *U6* for miRNA, respectively. The relative expression level of each RNA was calculated using 2^−ΔΔCt^ methods.

**Table 1.** The sequences of the used PCR primers.

Gene	Primers	Sequences (5′–3′)
*miR-15a-5p*	Forward	TAGCAGCACATAATGGTTTGTG
*miR-92a-3p*	Forward	TATTGCACTTGTCCCGGCCTGT
*miR-15b-5p*	Forward	TAGCAGCACATCATGGTTTACA
*miR-107*	Forward	AGCAGCATTGTACAGGGCTATCA
*miR-16-5p*	Forward	TAGCAGCACGTAAATATTGGCG
*miR-19b-3p*	Forward	TGTGCAAATCCATGCAAAACTGA
*miR-29b-3p*	Forward	TAGCACCATTTGAAATCAGTGT
*miR-19a-3p*	Forward	TGTGCAAATCTATGCAAAACTGA
*miR-144-3p*	Forward	TACAGTATAGATGATGTACT
For all *miRNA*	Reverse	CTCAACTGGTGTCGTGGA
*U6*	Forward	CTCGCTTCGGCAGCACA
	Reverse	ACGCTTCACGAATTTGCGTGTC
*GAPDH*	Forward	GCACCGTCAAGCTGAGAAC
	Reverse	TGGTGAAGACGCCAGTGGA
*XIST*	Forward	GCTCCAACCAATCTAAAAGG
	Reverse	TGCCCCATCTCCACCTAA
*CUL3*	Forward	GATGAGTTCAGGCAACATC
	Reverse	ATGTCTTGGTGCTGGTGG

### Statistical analyses

All data are expressed as the mean ± standard deviation (SD). Student’s *t*-test was used for differences between groups. A *p* value <.05 was considered statistically significant.

## Results

### Differential expression analysis

**Figure 1.**
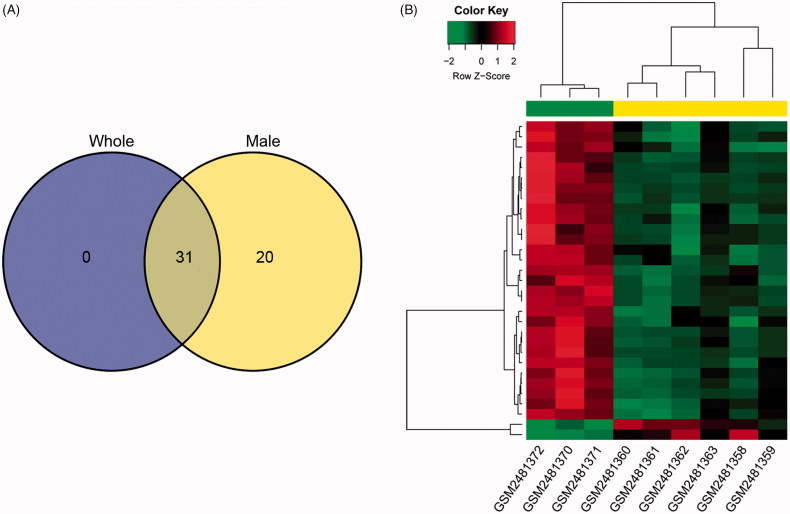
The Venn figure and bidirectional clustering heatmap for the differentially expressed miRNAs (DE-miRNAs). (A) The Venn figure of the DE-miRNAs between the whole AKI patients versus healthy controls and male AKI patients versus healthy controls; (B) The bidirectional clustering heatmap.

We firstly identified the DE-miRNAs from male patients (*n* = 4), and 51 DE-miRNAs, including 10 up-regulated and 41 down-regulated DE-miRNAs were identified versus controls (Figure 1(A)). With the mixing of two female samples (whole), 31 DE-miRNAs (two up-regulated and 29 down-regulated) were identified in sepsis-induced AKI group (male = 4, female = 2) compared with control group (male = 1, female = 2; Figure 1(A)), and all the 31 DE-miRNAs were common DE-miRNAs between the two comparisons. The bidirectional clustering heatmap of 31 common DE-miRNAs could clearly distinguish the samples in different groups (Figure 1(B)). We then focused on the function of the 31 common DE-miRNAs.

### MiRNA-target prediction and enrichment analysis

**Figure 2.**
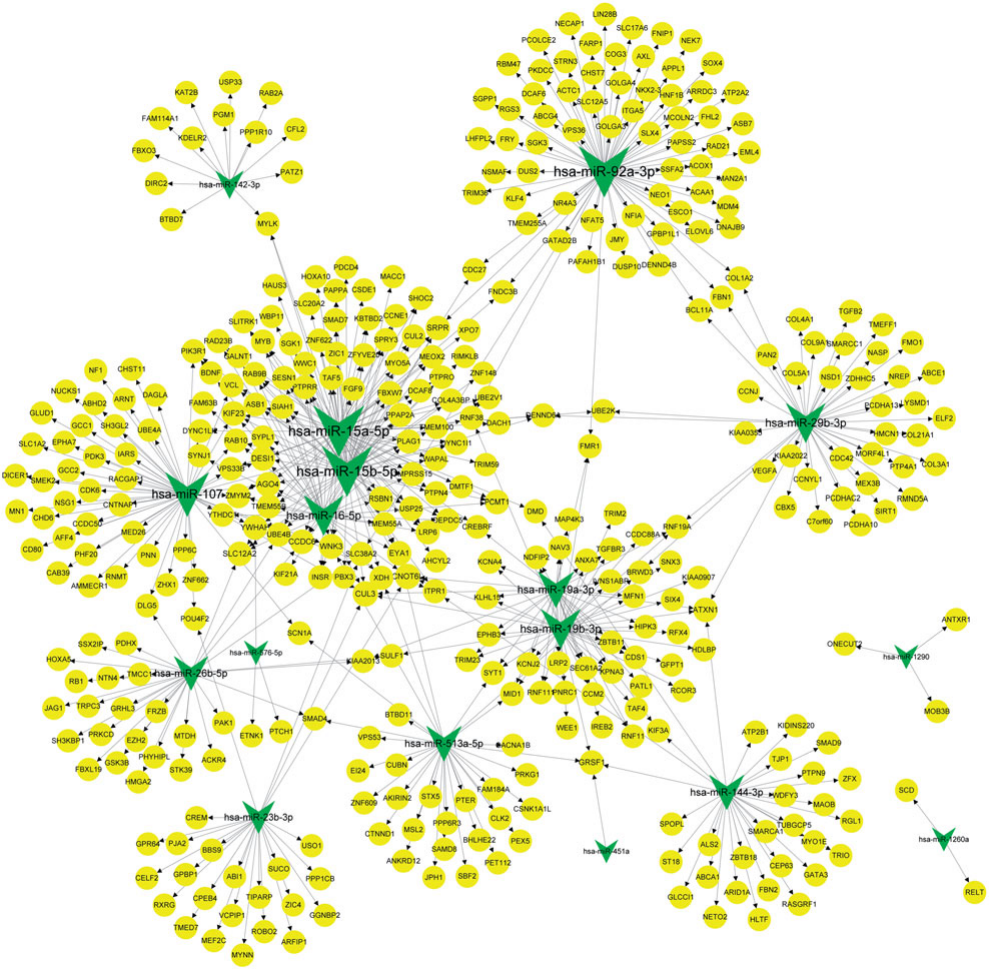
The miRNA-target regulatory network. Circles and arrows represent down-regulated miRNAs and target genes, respectively.

A total of 564 miRNA-target pairs (involving 17 down-regulated miRNAs) were predicted (Figure 2). The top 10 miRNAs with higher interaction degrees in the regulatory network are listed in Table 2.

**Table 2.** The top 10 miRNAs in the miRNA-target regulatory network.

miRNA	Description	Degree
*hsa-miR-15a-5p*	down	71
*hsa-miR-92a-3p*	down	71
*hsa-miR-15b-5p*	down	69
*hsa-miR-107*	down	53
*hsa-miR-16-5p*	down	43
*hsa-miR-19b-3p*	down	42
*hsa-miR-29b-3p*	down	42
*hsa-miR-19a-3p*	down	34
*hsa-miR-513a-5p*	down	32
*hsa-miR-144-3p*	down	31

**Figure 3.**
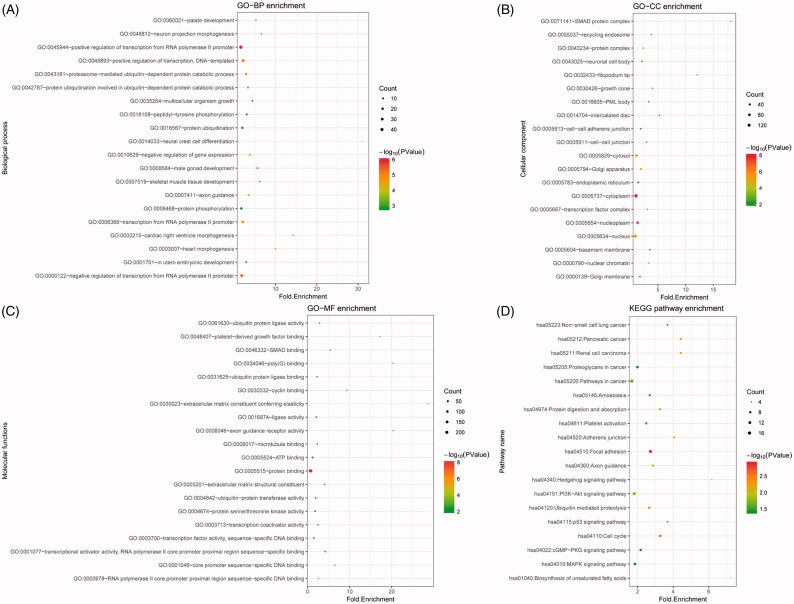
The top 20 terms enriched for the genes involved in the miRNA-target pairs. (A) The top 20 gene ontology (GO) biological process (BP) terms; (B) The top 20 GO cellular component (CC) terms; (C) The top 20 GO molecule function (MF) terms; (D) The top 20 Kyoto Encyclopedia of Genes and Genomes (KEGG) pathways. The deeper the color, the lower the *p* value is. The larger the circle, the more genes that were enriched.

**Figure 4.**
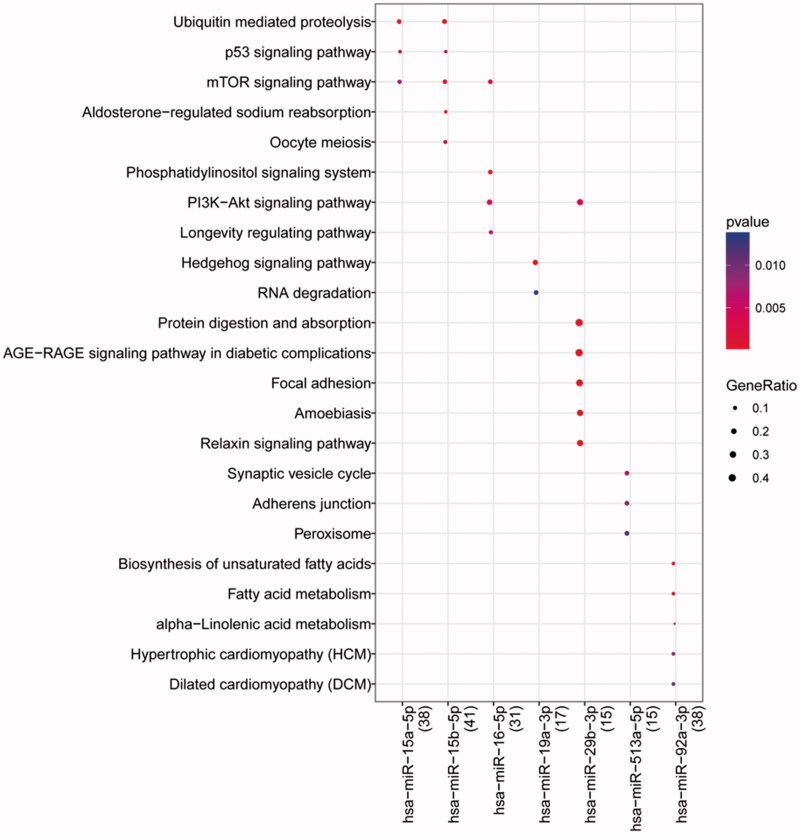
The pathway enriched for the top 10 miRNAs (top 5 shown). The deeper the color, the lower the p value is. The larger the circle, the more genes that were enriched.

Enrichment analysis showed that genes in the miRNA-target pairs were enriched into 111 GO_BP (such as positive regulation of transcription from RNA polymerase II promoter), 33 GO_CC (such as cytoplasm), and 43 GO_MF (such as protein binding), and 19 KEGG pathways (such as focal adhesion). The top 20 terms in each category are presented in Figure 3. Clusterprofiler analysis showed the top 10 miRNAs (down-regulated) were associated with 41 KEGG pathways, and the top 5 pathways enriched for each miRNA are shown in Figure 4. Especially, *miR-15a-5p*, *miR-15b-5p*, and *miR-16-5p* were involved in mTOR signaling pathway, and *miR-16-5p* and *miR-29b-3p* were enriched in PI3K-Akt signaling pathway.

### PPI network analysis for the target genes

**Figure 5.**
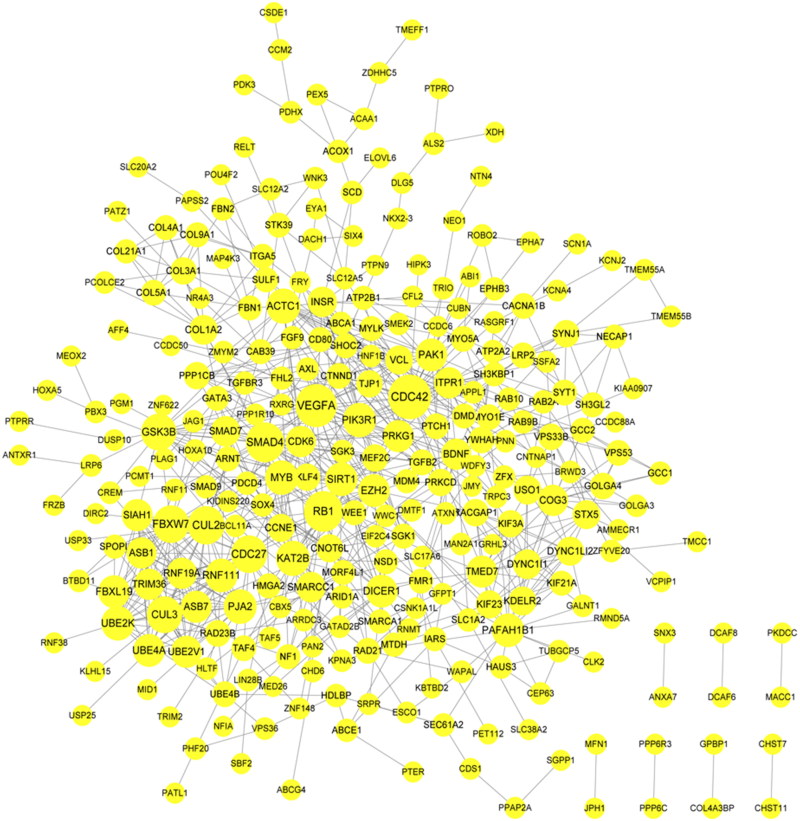
The protein-protein interaction (PPI) network for the target genes. The higher the degree value, the larger the node.

**Figure 6.**
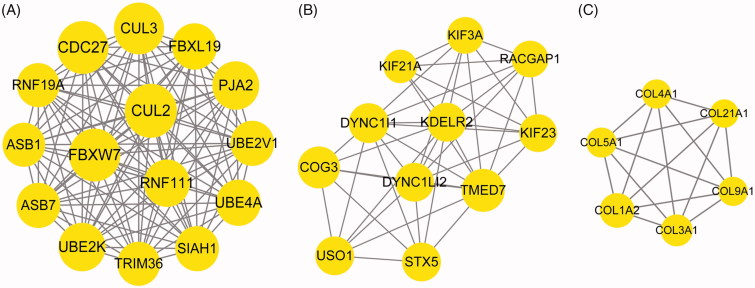
The significant modules identified from the protein-protein interaction (PPI) network. (A) The significant module A; (B) The significant module B; (C) The significant module C.

The PPI network for the target genes included 281 nodes (productions) and 776 edges (interactions; Figure 5). Three significant network modules (module A, score = 15, involving 15 nodes and 105 edges; module B, score = 8.2, involving 11 nodes and 41 edges; module C, score = 6, involving 6 nodes and 15 edges) were identified (Figure 6). The top 10 nodes in the PPI network and the nodes in the three significant network modules are listed in Table 3.

**Table 3.** The top 10 protein-protein interaction (PPI) network nodes and the module nodes.

Degree top 10		Module-A		Module-B		Module-C	
Nodes	Degree	Nodes	Degree	Nodes	Degree	Nodes	Degree
CDC42	31	CUL2	23	TMED7	15	COL1A2	12
VEGFA	29	CDC27	22	COG3	14	COL3A1	9
RB1	26	FBXW7	22	DYNC1I1	12	COL5A1	7
SMAD4	25	CUL3	21	STX5	11	COL9A1	6
CUL2	23	PJA2	18	USO1	11	COL4A1	6
FBXW7	22	UBE2K	18	DYNC1LI2	11	COL21A1	5
CDC27	22	RNF111	17	KDELR2	11		
GSK3B	21	UBE4A	16	KIF23	10		
CUL3	21	FBXL19	16	KIF3A	9		
KAT2B	19	SIAH1	15	RACGAP1	9		
		ASB7	15	KIF21A	6		
		UBE2V1	15				
		RNF19A	14				
		TRIM36	14				
		ASB1	14				

### CeRNA regulatory network analysis

**Figure 7.**
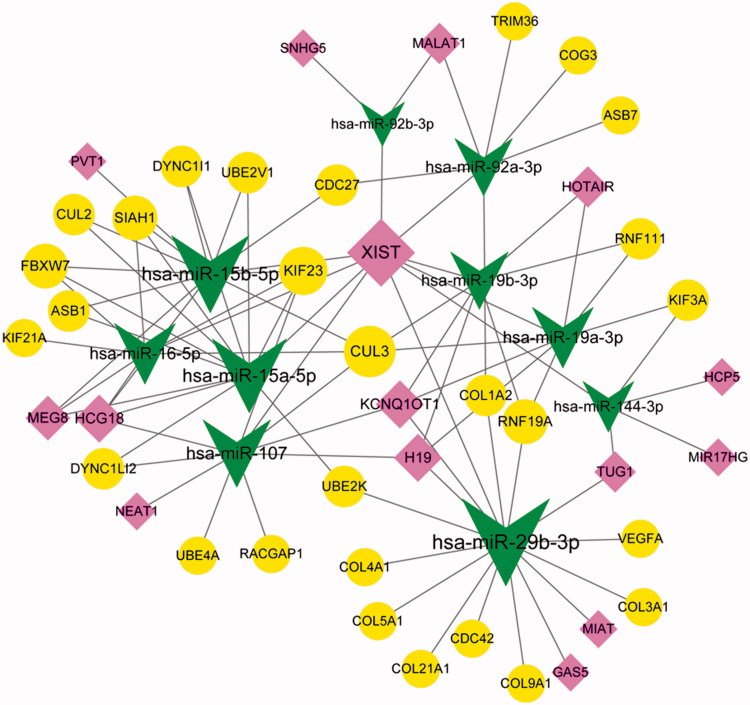
The competing endogenous RNA (ceRNA) regulatory network. Arrows, circles, and diamonds represent down-regulated miRNAs, target genes, and long non-coding RNAs (lncRNAs), respectively. The higher the degree value, the larger the node is.

For the top 10 miRNAs in the miRNA-target regulatory network, 38 miRNA-lncRNA pairs were predicated. After integrating the above mentioned miRNA-lncRNA pairs and the miRNA-target pairs, 89 miRNA-lncRNA-mRNA regulatory pairs (involving 10 down-regulated miRNAs, 15 lncRNAs, and 28 mRNAs) were obtained (Figure 7), and the nodes with top 10 degrees (including *miR-29b-3p*; *miR-15b-5p*; *miR-15a-5p*; *miR-107*; X inactive specific transcript, *XIST*; *miR-19a-3p*; *miR-16-5p*; *miR-19b-3p*; *miR-92a-3p*; and Cullin 3, *CUL3*) are listed in Table 4. Importantly, the *miR-15b-5p/miR-16-5p/miR-19a-3p/miR-19b-3p/miR-107-XIST-CUL3* regulatory axis involving the top 10 nodes existed in the ceRNA regulatory network.

**Table 4.** The top 10 nodes in the competing endogenous RNA (ceRNA) regulatory network.

Nodes	Description	Degree
*hsa-miR-29b-3p*	miRNA	16
*hsa-miR-15b-5p*	miRNA	13
*hsa-miR-15a-5p*	miRNA	12
*hsa-miR-107*	miRNA	10
*XIST*	lncRNA	10
*hsa-miR-19a-3p*	miRNA	8
*hsa-miR-16-5p*	miRNA	8
*hsa-miR-19b-3p*	miRNA	7
*hsa-miR-92a-3p*	miRNA	7
*CUL3*	gene	5

lncRNA: long non-coding RNA.

### Validation of the downregulation of RNA expression in AKI samples and in vitro LPS-induced cellular AKI model

**Figure 8.**
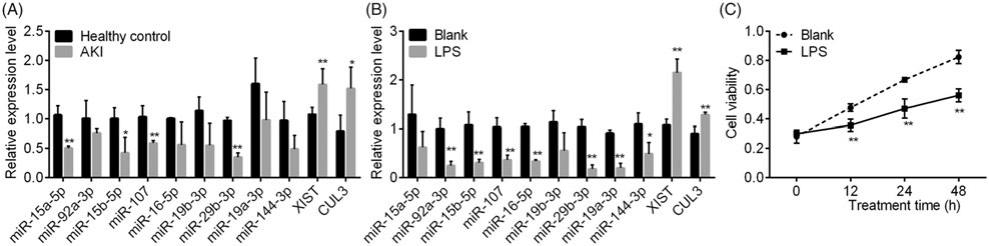
The validation experiments. A and B, the qPCR analysis for several differentially expressed miRNAs in five patients with AKI (male = 4 and female = 1) versus healthy controls (sex and age-matched), and that in LPS-induced cellular AKI model. C, the cell viability by CCK8 assay in MPC5. *p < .05 vs. blank. **p < .01 vs. blank. LPS: lipopolysaccharide.

The validation using AKI samples confirmed the significant downregulations of *miR-15a-5p*, *miR-15b-5p*, *miR-107*, *miR-29b-3p* and the upregulation of *XIST* and *CUL3* (Figure 8(A)). The downregulation of *miR-92a-3*p, *miR-16-5*p, *miR-19a-3*p, and *miR-144-3*p were not significant in AKI samples versus controls (*p* > .05). In the *in vitro* validation experiments in MPC5, we found LPS treatment significantly decreased the relative expression of 7 of 9 miRNAs, including *miR-92a-3p*, *miR-15a-5p*, *miR-107*, *miR-16-5p*, *miR-29a-3p*, *miR-19a-3p* and *miR-144-3p* (*p* < .05, Figure 8(B)), and inhibited the proliferation of MPC5 (*p* < .01, Figure 8(C)). The expression of *XIST* and *CUL3* were obviously upregulated by LPS (*p* < .01).

### The confirmation of XIST-miR-15a-5p-CUL3 ceRNA axis

**Figure 9.**
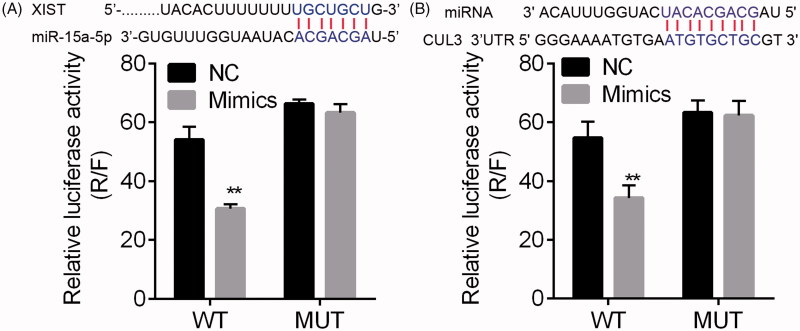
The results of dual-luciferase reporter assay. The interaction between lncRNA and miRNA (A) and between miRNA and target (B) were predicated using the LncBase Predicted v(0).2 and miRTarBase, respectively. The interaction was validated using dual-luciferase reporter system. **p < .01 vs. NC.

**Figure 10.**
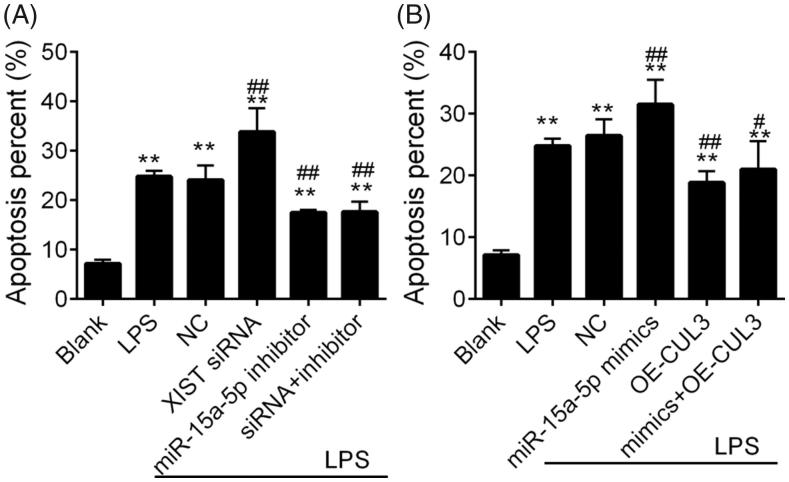
The apoptosis of MPC5 cells. A and B, the validation of XIST/miR-15a-5p axis and miR-15a-5p/CUL3 axis on cell apoptosis, respectively. **p < .01 vs. Blank. # and ## notes *p* < .05 and .01 vs. LPS, respectively. LPS: lipopolysaccharide.

The interactions of *XIST/miR-15a-5p* and *miR-15a-5p/CUL3* were predicted and then validated using the dual-luciferase reporter assay systems (Figure 9(A,B)). This suggested the dysregulated *miR-15a-5p*-*XIST*-*CUL3* ceRNA axis during AKI. The apoptosis analysis using the transfected MPC5 cells showed that the inhibition of *XIST* and *miR-15a-5p* respectively enhanced and reserved LPS-induced apoptosis significantly (Figure 10(A) and Figure S1), while the *miR-15a-5p* inhibitor reversed *XIST* siRNA enhanced MPC5 cell apoptosis. In addition, the overexpression of *CUL3* significantly reduced LPS-induced apoptosis, and showed inhibitory effect on *miR-15a-5p* mimics induced apoptosis (Figure 10(B)). These data revealed the *XIST*-*miR-15a-5p*-*CUL3* axis.

## Discussion

In this study, 31 DE-miRNAs (two up-regulated and 29 down-regulated) were identified in blood samples from patients (two females and four males) with sepsis-induced AKI compared with healthy controls. This was different from the 37 DE-miRNAs by Ge et al by compared with sepsis-induced AKI and sepsis-non AKI [27]. However, some miRNAs were also identified to be downregulated in sepsis-induced AKI versus sepsis-non AKI, like *miR-15b-5p*, *miR-15a-5p* and *miR-16-5p*. We identified several key miRNAs, including *miR-15a-5p*, *miR-15b-5p*, and *miR-16-5p* were involved in mTOR signaling pathway, and *miR-16-5p* and *miR-29b-3p* were associated with PI3K-Akt signaling pathway, the similar pathways identified by Ge et al. These two pathways have been reported to be impressive in ischemia-reperfusion (I/R)-induced AKI [28]. Additionally, *miR-29b-3p*, *miR-15b-5p*, *miR-15a-5p*, *miR-107*, *XIST*, *miR-19a-3p*, *miR-16-5p*, *miR-19b-3p*, *miR-92a-3p*, and *CUL3* were the top 10 nodes in the ceRNA regulatory network. Therefore, the significant downregulation of these miRNAs might suggest the important roles of them in sepsis-induced AKI via involving mTOR and PI3K-Akt signaling pathways.

The overexpression of *miR-29a* was found to be independent risk factor for the mortality of patients with septic AKI, and thus they can be applied for predicting the 28-day mortality of the disease [29]. Huo et al reported that the expression level of *miR-29a* were positively correlated with the levels of serum creatinine, cystatin C, and kidney injury molecule-1 (KIM-1) in patients with septic AKI. However, Drummond et al reported that *miR-29a-3p* was increased in the kidney of mice exposed to e-cigarette air. The expression of its targets including Collagen 1A1 and Fibrillin 1 as well as renal fibrosis was significantly increased [30]. Overexpressed *miR-107* causes TNF-α secretion through regulating dual-specificity phosphatase 7(*DUSP7*) in circulating endothelial cells, which may lead to tubular cell damage in sepsis-induced AKI [31]. Our present study demonstrated that the *DUL3* is a predictive target of *miR-107* and was upregulated by LPS treatment in MPC5. CUL3 is part of the ubiquitin proteasomal system and the *Cul3* disruption is associated with tubulointerstitial fibrosis [32]. Saritas *et al* reported that the upregulation of *CUL3* in patients with AKI and *Cul3* disruption increased the expression of cyclin E and p21 and the promotion of proximal tubule injury in mice [32]. In addition, other studies identified the elevation of *miR-92a-3p* in chronic kidney injury (CKD) and CKD-associated atherosclerosis [33,34]. These suggested that *miR-29b-3p*, *miR-107*, and *miR-92a-3p* might be correlated with the pathogenesis of sepsis-induced AKI. The specific mechanism mediated by them should be explored.

The downregulation of *miR-15a-5p* has been identified in CKD with hypertension [35,36]. In addition, Ge et al suggested the downregulation of *miR-16-5p*, *miR-15a-5p* and *miR-15b-5p* in sepsis-induced AKI versus sepsis non AKI [27]. This consistency showed the important roles of these miRNAs in AKI. The difference is our present study identified lncRNAs related to these miRNAs. The expression of lncRNAs *XIST* and *NEAT1* are significantly increased in glomerular and tubular epithelial cells, and urinary *XIST* serves as a potential marker for detecting membranous nephropathy [37]. Elevated *XIST*, which could be induced by LPS, is correlated with glomerular nephritis [37]. Our *in vitro* experiment using LPS-treated MPC5 cells showed *XIST* was elevated by LPS stimulus. We identified *XIST* regulated the expression of upregulated *CUL3* by sponging the top 10 downregulated miRNAs, including *miR-15a-5p, miR-16-5p* and *miR-107*. The upregulation of *CUL3* has been identified in patients with AKI [32]. Mutations in *CUL3* reduced ubiquitination in the kidneys [38,39]. These studies indicated that *XIST* plays impressive roles in AKI via regulating *CUL3* by sponging miRNAs. The *miR-15a-5p-XIST-CUL3* regulatory axis in the ceRNA regulatory network was validated using the *in vitro* cellular experiment. We also demonstrated that *miR-15a-5p* inhibition reserved MPC5 cell apoptosis that was enhanced by *XIST* siRNA, so did as the overexpression of *CUL3* for *miR-15a-5p* mimics-induced apoptosis in MPC5 cells. These data suggested that *miR-15a-5p-XIST-CUL3* played crucial roles in sepsis-induced AKI, and the management targeting this axis might be of great value for controlling AKI development.

## Conclusions

In conclusion, we identified 31 DE-miRNAs in sepsis-induced AKI samples compared with healthy control. Among them, *miR-29b-3p*, *miR-15b-5p*, *miR-15a-5p*, *miR-107*, *XIST*, *miR-19a-3p*, *miR-16-5p*, *miR-19b-3p*, *miR-92a-3p*, and *CUL3* were closely related to the pathogenesis of sepsis-induced AKI. Moreover, the *miR-15a-5p-XIST-CUL3* ceRNA regulatory axis was involved in the mechanism of sepsis-induced AKI and the LPS-induced injury in MPC5 cells.
